# Suicidal risk and psychopathological profiles in adolescents with neurodevelopmental disorders: an Italian multicentric study

**DOI:** 10.3389/fpsyt.2025.1614270

**Published:** 2025-08-15

**Authors:** Ramona Cardillo, Irene Di Modica, Francesca Cucinotta, Federica Galletta, Alessia Raffagnato, Marcella Di Cara, Carmela De Domenico, Eva Germanò, Sara Carucci, Giuseppe Abbracciavento, Rita Murtas, Alessia Donia, Giuliana Bonelli, Serenella Grittani, Evamaria Lanzarini, Arianna Accetta, Martina Pirrone, Carola Costanza, Rosamaria Siracusano, Clemente Cedro, Michela Gatta, Antonella Gagliano

**Affiliations:** ^1^ Department of Developmental Psychology and Socialization, University of Padua, Padua, Italy; ^2^ Child and Adolescent Neuropsychiatry Unit, Department of Women’s and Children’s Health, Padua University Hospital, Padua, Italy; ^3^ Child and Adolescent Neuropsychiatry Unit, Department of Human and Evolutionary Pathology “Gaetano Barresi”, University of Messina, Messina, Italy; ^4^ Istituto di Ricovero e Cura a Carattere Scientifico (IRCCS) Centro Neurolesi Bonino Pulejo, Department of Bioparco delle intelligenze e delle neurofragilità, Messina, Italy; ^5^ Child and Adolescent Neuropsychiatry Unit, Department of Medical Sciences and Public Health, University of Cagliari and “A Cao” Pediatric Hospital, Cagliari, Italy; ^6^ Department of Clinical and Experimental Medicine, University of Messina, Messina, Italy; ^7^ Department of Mental Health, Center for Children with Autism Spectrum Disorder (ASD), Rimini, Italy; ^8^ Child and Adolescent Neuropsychiatry Unit, Infermi Hospital, Rimini, Italy; ^9^ Department of Psychology, Educational Science and Human Movement, University of Palermo, Palermo, Italy; ^10^ Division of Child Neurology and Psychiatry, University Hospital Federico II, Napoli, Italy; ^11^ Department of Biomedical and Dental Sciences and Morphofunctional Imaging, University of Messina, Messina, Italy; ^12^ Psychiatry Unit, Polyclinic Hospital, University of Messina, Messina, Italy; ^13^ Child and Adolescent Neuropsychiatry, Department of Medicine and Surgery, “Kore” 90 University of Enna, Enna, Italy; ^14^ Department of Neuroscience, Oasi Research Institute-IRCCS, Troina, Italy

**Keywords:** suicidal spectrum behaviors, neurodevelopmental disorders, pre-adolescents, adolescents, emotional dysregulation, adverse life events

## Abstract

**Introduction:**

Suicidal spectrum behaviors (SSB) consist of a continuum ranging from non suicidal self-injury, suicidal ideation, and suicide attempt to committed suicide. In adolescence, suicide is currently the second cause of death among adolescents aged 15 to 24 years and the third leading cause in children aged 10 to 14 years. Adolescents with Neurodevelopmental Disorders (NDDs), especially those with Autism Spectrum Disorder and Attention Deficit/Hyperactivity Disorder, are at heightened risk.

**Methods:**

This is a cross-sectional, multicenter research study, which involves four Italian child neuropsychiatry units (Messina, Padua, Rimini, and Cagliari). The study aims to define a specific neuropsychological and psychopathological profile associated with suicidal behaviors in adolescents and pre-adolescents with NDDs (11–18 years). In a sample of 127 NDDs adolescents (60 females and 67 males), with and without SSB, several variables were compared through standardized measures, including emotional dysregulation, impulsivity, and irritability as well as environmental risk factors.

**Results:**

The results of this study are consistent with the literature data and suggest that emotional dysregulation (p <.001) as an individual factor and Adverse Childhood Experiences (p = .002), as environmental factors, play a key role in promoting suicidality in pre-adolescents and adolescents with NDDs.

**Discussion:**

This awareness prompts the implementation of useful prevention methods during the clinical follow-up of individuals with NDDs.

## 1 Introduction

According to extremely concerning data, suicide ranks as the third cause of mortality for children aged 10 to 14 and the second cause of death for adolescents aged 15 to 24 (1). Furthermore, it is now recognized that data on the number of suicides are often significantly underestimated due to social and cultural stigma and its legal implications (2). Considering these alarming data, it becomes clear that suicide and self-harm in young people are serious public health problems. According to the World Health Organization (WHO) (3) the presence of self-harming behaviors—whether non-suicidal or suicidal—substantially increases the risk of suicide. Therefore, it is more appropriate to conceptualize this phenomenon as suicidal spectrum behaviors (SSB), which encompass a continuum ranging from non-suicidal self-injury (NSSI) to suicidal ideation, suicide attempts (SA), and ultimately, completed suicide (4).

Current evidence shows that psychiatric and psychological conditions represent three of the ten most frequently identified risk factors for self-harming behavior, according to general literature reviews (5).

Among psychiatric conditions, depressive disorder, bipolar disorder, psychosis, and substance abuse have traditionally been deeply studied (6). However, the rate of SSB appears particularly high also in Neurodevelopmental Disorders (NDDs), mainly in attention-deficit/hyperactivity disorder and autism spectrum disorder (7). NDDs represent a heterogeneous group of conditions characterized by developmental deficits that cause impairment of personal, social, academic and occupational functioning. According to the DSM-5 (8) the diagnostic class of neurodevelopmental disorders includes Intellectual disability (ID), Language and communication disorders (CD), Autism Spectrum Disorder (ASD), Attention-Deficit/Hyperactivity Disorder (ADHD), Specific Learning Disorders (SLD) and movement disorders, including Tourette Syndrome (TS). The literature on risk factors for self-harm behavior and suicide in children and adolescents with NDDs, reveals a broad body of research focused on adolescents with ADHD, ASD, and TS but a relative paucity of information on ID, CD, and SLD (9). Even though research on SSB in NDDs has recently increased, literature data remain limited, and studies conducted so far appear insufficient in identifying the risk factor profile for SSB in NDDs.

Attention-Deficit/Hyperactivity Disorder increases the risk of facing mental health challenges, social difficulties, suicide, and premature mortality, mostly during the transition into adulthood (10). A recent systematic review shows that several demographic and clinical features are associated with an increased risk of SSBs in adolescents and adults with ADHD (11). The rate of suicide attempts seems to be higher in females (ratio: 1:4) than in males (ratio: 1:7), with females presenting a higher prevalence of associated mood disorders. The research focused on examining the relationship between ADHD symptoms presentation and SSBs described the combined presentation as the higher risk factor for self-harm behaviors, suicidal ideation, and suicide attempt (12, 13). Nevertheless, both inattention and hyperactivity/impulsivity symptom severity scores represent childhood predictors of NSSI and SA (12), supporting the hypothesis that overall ADHD symptom severity is an important factor concerning SSBs.

Autism Spectrum Disorder is characterized by persistent communication and social interaction difficulties and restricted and repetitive behaviors, which can vary in severity. Recently, some studies attest a higher mortality rate of ASD individuals, even double that of the general population, describing suicide as one of the potential causes of death (14). Meanwhile, a few population studies have tried to determine the incidence of suicide deaths in ASD patients, rated around 7,7% for patients who committed suicide and 30,8% for patients that presented suicidal ideation (15, 16). Self-injurious behavior, suicidal thoughts, and suicide attempts are also described as more common in ASD patients than in the general population (17, 18). Associated conditions, as ADHD symptoms, significantly increase the risk of suicidal behaviors (19). However, the social communication difficulties due to autistic traits remain an inherent risk for suicidality, so much so that individuals with ASD associated with severe social communication difficulties appear more at-risk of attempting suicide, planning suicide, and have suicidal thoughts, but not self-harm without suicidal intent (20).

Intellectual Disability is characterized by deficits in intellectual and adaptive functioning in the conceptual, social, and practical areas of life during the developmental period (8). For a long time, it was assumed that the presence of ID may act as a protective factor against suicide due to the lack of cognitive sophistication to conceptualize, plan, or commit suicide (21–23). However, in recent literature, individuals with ID have been described as perfectly capable of developing an awareness of their suffering and considering suicide as an escape route. It should be noted, however, that suicidal thoughts and attempts are more common in people with mild intellectual disability and less frequent in people with severe-moderate disability (24, 25). This underlines the fact that both the self-reflective and the design functions are necessary pre-requisites for implementing anti-conservative actions.

Specific learning disorder concerns the impairment of specific skills, such as the speed and accuracy of reading words, spelling, and mathematical calculation. Suicidal ideation and attempts are more frequent in students with reading difficulties than in students with advanced reading skills (26). When not diagnosed promptly, SLD can interfere with academic learning and career success and affect daily activities and social interactions (8, 27). Suicidality can also be related to resignation, characterized by a sense of helplessness due to traumatic events or persistent failure (28). This depressive form, defined as “learned helplessness,” is described as one of the causes supporting depressive pictures in subjects with SLD (29). In summary, SLD and related school trauma predispose the subject to suicide through an increase in the likelihood of developing depression and, consequently, self-injurious behaviors and suicidality. A growing body of literature suggests a bidirectional relationship between SLD and depression. On one hand, persistent academic difficulties, repeated failure, and negative social comparison can undermine self-esteem and increase vulnerability to depressive symptoms (30). On the other hand, depression itself can impair cognitive processes such as attention, working memory, and executive functioning, further worsening learning performance and reinforcing a vicious cycle (31).

Communication disorders include language disorder, phonetic-phonological disorder, verbal fluency disorder, and language pragmatics disorder with early developmental onset (8). These disorders can have different levels of severity and can coexist with each other (32). Moreover, CD may impact the individual’s ability to recognize and express emotions, leading to difficulties in emotional processing and regulation. Early intervention for communication disorders seems to play a protective role in enhancing emotion regulation skills (33). Thus, improving emotional states in individuals with CD benefits in preventing suicide at-risk behaviors (34). Nevertheless, the paucity of research on SSB in this disorder doesn’t allow us to draw solid conclusions.

Tourette syndrome is a childhood-onset neurodevelopmental disorder characterized by multiple motor and vocal tics present for at least one year. The risk of suicide is considerably high in individuals with TS. In a large cohort study of 7736 individuals with TS/CTD from the Swedish National Patient Register, individuals with TS compared with control subjects have an increased risk of both dying by suicide and attempting suicide (35). Furthermore, literature data highlight that the majority of individuals with TS present concomitant behavioral problems, most commonly obsessive-compulsive disorder (OCD) and ADHD (36, 37), which in themselves increase the suicide risk. The persistence of tics beyond young adulthood and previous suicide attempts are the strongest predictors of death by suicide in individuals with TS (38).

The aim of this study is to analyze specific neuropsychological and psychopathological factors potentially associated with SSB in adolescents and pre-adolescents with NDDs (11–18 years). Various features, including psychiatric and psychological dimensions and particularly emotional dysregulation, impulsivity, hopelessness, and pessimism, have been identified as potential risk factors for suicidal behavior. However, they have not yet been specifically analyzed in the context of NDDs. It must be considered that the psychological structure of individuals with neurodevelopmental disorders is inevitably shaped by the limits and the difficulties linked to the NDDs and to their influence on learning and life experiences. Thus, the evaluation of samples of individuals with NDDs is crucial for a deeper knowledge of the risk factors for SSB that could be specific for this population. The study aims to take into account factors such as emotion regulation, emotion recognition, internalizing and externalizing problems, impulsivity, and adverse life events in order to describe the psychological characteristics potentially associated with SSB. Improving the awareness of these characteristics can guide the development and the implementation of effective prevention strategies and protective factors that may, in turn, reduce the likelihood of self-harming behaviors in individuals with NDDs.

## 2 Materials and methods

### 2.1 Study design

This multicenter cross-sectional observational study was conducted in five Italian Child and Adolescent Neuropsychiatry Units (University Hospital of Messina; University Hospital of Padua; NPIA ASP Rimini; “A. Cao” Pediatric Hospital Cagliari; IRCCS Centro Neurolesi Bonino Pulejo, Messina) between July and December 2024. The study protocol was approved by the Ethical Committee of the University of Messina (Messina, Italy) in July 2024 (prot. n. 124/24) and was conducted in accordance with the ethical principles of the Helsinki Declaration for medical research involving human subjects. All psychological and diagnostic evaluation were performed by qualified and experienced clinical psychologists and child neuropsychiatrists at each center.

### 2.2 Participants

Consecutively, outpatients or inpatients from the five Child and Adolescent Neuropsychiatry Units were enrolled. The inclusion criteria were: a) age between 11 and 18 years old at the time of enrollment; b) diagnosis of NDDs (ADHD, ASD, ID, CD, LSD, TS) according to the DSM-5; c) Full-scale intelligence quotient >60; d) fluent language skills, with the ability to use both simple and complex sentences. Patients with a diagnosis of moderate or severe intellectual disability were excluded, as well as patients with genetic syndrome or other severe neurological conditions (e.g. cerebral palsy, epilepsy). The Italian version of the Wechsler Intelligence Scale for Children, fourth edition (39), has been used to assess the full-scale intelligence quotient. Specifically, the Verbal Comprehension item of WISC IV was used to evaluate language skills.

Participants were enrolled after obtaining informed consent from the participants and/or their legal guardians.

### 2.3 Suicide-risk behavior assessment

The sample was divided into two groups based on the presence of suicide-risk behaviors, as assessed using the Columbia-Suicide Severity Rating Scale (C-SSRS) (40). The C-SSRS is a tool used to assess the severity and immediacy of suicide risk. It considers several aspects, including.

− Suicidal Ideation (SI): frequency, duration, and intensity of suicidal thoughts.
− Suicidal Behavior (SB): any previous suicide attempts, preparations, or acts of non-suicidal self-harm.
− Intensity of Suicidal Ideation: a detailed assessment of the nature and severity of suicidal thoughts.

The C-SSRS screener is comprised of between 2–6 self-reported “yes” or “no” questions. Affirmative responses count as 1 point, which are then summed to indicate the level of suicide risk on a scale of 0 - 6. The cut-off for considering a patient at-risk is a positive score (1 – 2 = Low risk; 3 – 6 = Moderate to high risk) to one of the items SI and SB in C-SSRS, while no-risk groups don’t have positive scores for those items (0 = no-risk reported).

### 2.4 Psychopathological assessment

In order to evaluate psychopathological factors, participants filled out a battery of standardized scales (both self-report and clinician-administered). These measures were selected based on specific risk factors for suicidal spectrum behaviors reported in the literature (8).

• Youth Self Report for Ages 11-18 (YSR) (41); is a self-report survey that uses 112 questions to evaluate behavioral issues and emotional functioning in adolescents aged 11 to 18 years, providing a detailed profile of the adolescent’s problems and competencies. Some of the main scales include Internalizing Problems, Externalizing Problems, Syndrome Scales, Competence Scale, and DSM-Oriented Scales.

− Child Behavior Checklist for Ages (CBCL/6-18) (41): is a psychological assessment used to measure behavioral problems and social competencies in children and adolescents aged 6 to 18 years. The CBCL/6–18 consists of two main sections: Competence Scales (social, academic, and activity functioning), and Problem Scales, which measure various behavioral and emotional problems through specific items. However, parents filled out the Child Behavior Checklist for Ages 6-18 (CBCL/6-18), the Four Factor Index of Social Status (SES) (42) questionnaire, and a demographic form with anamnestic data.
− Difficulties in Emotion Regulation Scale – Short Form (DERS-SF) (43): is a psychometric tool used to assess difficulties in emotional regulation that may be associated with various neuropsychiatric disorders. It evaluates six different dimensions of emotional regulation: Non-acceptance of Emotional Responses, Difficulties Engaging in Goal-Directed Behavior, Impulse Control Difficulties, Lack of Emotional Awareness, Limited Access to Emotion Regulation Strategies, and Lack of Emotional Clarity.
− Toronto Alexithymia Scale (TAS-20) (44, 45): is a self-report tool consisting of 20 items, designed to measure the level of alexithymia in individuals.
− Barratt Impulsiveness Scale (BIS-11) (46–48): is a self-report psychometric tool used to measure impulsivity levels in an individual. It consists of 30 items, divided into three main factors, each representing a distinct dimension of impulsivity: Attentional Impulsiveness, Motor Impulsiveness, and Non-Planning Impulsiveness.
− Yale-Vermont Adversity in Childhood Scale (Y-VACS) (49): is a psychological assessment tool developed to measure adverse childhood experiences. It focuses on identifying events and conditions (for example, trauma, abuse, neglect, family difficulties) that may have a long-term impact on mental health and psychological well-being.

### 2.5 Statistical analysis

Statistical analyses were performed using R (50). A series of univariate ANOVAs were conducted to examine similarities and differences between the two groups (at-risk and no-risk NDDs) on continuous variables. The assumption of homogeneity of variances was tested using Levene’s test. In cases where this assumption was violated, Welch’s correction was applied. Effect sizes were calculated using Cohen’s d, which quantifies the magnitude of the difference between two means. Additionally, a cross-tabulation was performed, and the χ² test was used to assess statistically significant differences in the distribution of nominal variables across groups.

## 3 Results

The final sample consisted of 127 participants, including 60 females and 67 males. Analysis of neurodevelopmental diagnoses revealed that many participants had multiple coexisting conditions. Specifically, n. 4 participants had communication disorders, n. 67 were diagnosed with ADHD, n. 41 with ASD, n. 41 SLD, n. 12 presented tics/TS, and n. 5 had a mild intellectual disability. Since several individuals had more than one diagnosis, the total count of conditions exceeds the number of participants.

### 3.1 Sociodemographic characteristics

The sociodemographic characteristics of the sample are shown in **Table 1**, stratified by NDDs at-risk status. A significant age difference was observed between the two groups (F (1,126) = 15.452, p <.001), with the NDDs “at-risk group” being older on average than the no-risk group.

**Table 1:** Comparison of sociodemographic variables between NDDs at-risk and no-risk groups.

Variable	N	NDDs at-risk (N=53) % (N)	NDDs no-risk (N=74) % (N)	Test Statistics	*p*
Age, M (SD)	127	15.28 (1.77)	13.96 (1.94)	F (1, 126) = 15.452	**<.001**
Gender				χ² (1) = 8.233	**.004**
Male (M)	67	37.7 (20)	63.5 (47)		
Female (F)	60	62.3 (33)	36.5 (27)		
Region of Residence				χ² (3) = 8.569	**.036**
Sicily	47	24.5 (13)	45.9 (34)		
Sardinia	33	26.4 (14)	25.7 (19)		
Emilia-Romagna	15	18.9 (10)	6.8 (5)		
Veneto	32	30.2 (16)	21.6 (16)		
SES				χ² (4) = 1.472	.832
Low	31	25.0 (13)	25.4 (18)		
Low-Medium	26	17.3 (9)	23.9 (17)		
Medium	16	11.5 (6)	14.1 (10)		
Medium-High	37	34.6 (18)	26.8 (19)		
High	13	11.5 (6)	9.9 (7)		
Immigration Status	40.94	15.4 (8)	84.6 (44)	χ² (1) = 1.637	.20
Parental Marital Status				χ² (3) = 5.221	.15
Married/Co-habiting	84	66.7 (34)	69.4 (50)		
Separated	31	25.5 (13)	25.0 (18)		
Parent Deceased	5	7.8 (4)	1.4 (1)		
Adoption	3	0	4.2 (3)		

The values presented in bold indicate statistically significant effects. SES, Socioeconomic Status (42).

Gender distribution also varied significantly (χ² (1) = 8.233, p = .004), with a higher proportion of females in the NDDs “at-risk group” (62.3%) compared to the “no-risk group” (36.5%). Conversely, males were more prevalent in the “no-risk group” (63.5%) than in the “at-risk group” (37.7%).

Regarding region of residence, a significant association was found between geographic location and NDD risk status (χ² (3) = 8.569, p = .036). Participants from Emilia-Romagna were more frequently in the NDD “at-risk group” (18.9%) than in the “no-risk group” (6.8%). In contrast, individuals from Sicily were predominantly in the “no-risk group” (45.9%) compared to the “at-risk group” (24.5%). No significant differences were found between groups for participants from Sardinia and Veneto.

Socioeconomic status (SES) did not differ significantly between the two groups (χ² (4) = 1.472, p = .832). The distribution across SES categories was similar, with the highest proportion of participants in both groups falling into the medium-high SES category (34.6% in the risk group and 26.8% in the no-risk group). Finally, no significant differences were found in immigration status between the two groups (χ² (1) = 1.637, p = .20) or in terms of parental marital status (χ² (3) = 5.221, p = .15).

### 3.2 Family and individual medical history

**Figure 1:**
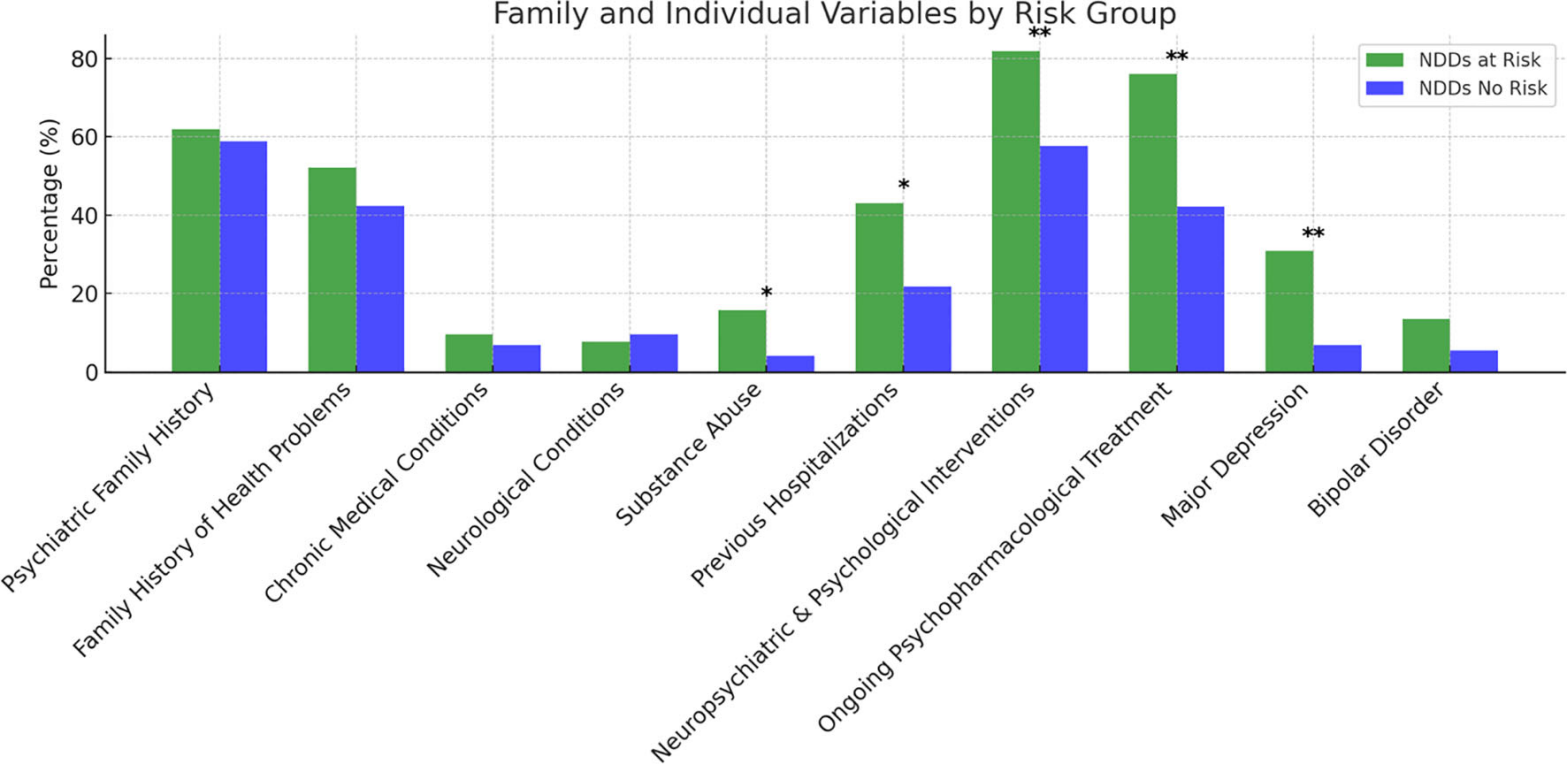
Family and Individual Variables by Risk Group (NDDs at-risk *vs* no-risk). Statistical significance is indicated as follows: *p < 0.05 (significant) **p < 0.01 (highly significant).

**Figure 1** presents family and individual variables by NDDs at-risk status. No significant differences were found between the two groups in terms of psychiatric family history (χ² (1) = 0.107, p = .744), family history of health problems (χ² (1) = 0.958, p = .328), chronic medical conditions (χ²(1) = 0.316, p = .574), or neurological conditions (χ²(1) = 0.136, p = .712).

However, significant differences emerged in other individual variables. Participants in the NDDs “at-risk group” reported a higher prevalence of substance abuse (15.7%) compared to the “no-risk group” (4.1%), χ² (1) = 5.090, p = .024. Similarly, previous hospitalizations were significantly more frequent among the NDDs “at-risk group” (43.1%) than in the “no-risk group” (21.7%), χ² (1) = 6.296, p = .012.

Neuropsychiatric and Psychological Interventions also differed significantly between groups, with a higher proportion of individuals in the NDDs “at-risk group” having received psychological or neuropsychiatric care (82.0%) compared to the “no-risk group” (57.6%), χ² (1) = 7.812, p = .005. Additionally, ongoing psychopharmacological treatment was significantly more common in the NDDs “at-risk group” (76.0%) than in the “no-risk group” (42.2%), χ² (1) = 13.095, p <.001.

Regarding comorbid psychiatric diagnoses, major depression was significantly more prevalent in the NDDs “at-risk group” (30.8%) than in the “no-risk group” (6.8%), χ² (1) = 12.431, p <.001. Although bipolar disorder was more frequently reported in the NDDs at-risk group (13.5%) than in the “no-risk group” (5.5%), this difference did not reach statistical significance (χ² (1) = 2.411, p = .12).

These results are shown in **Supplementary Table S1**.

### 3.3 Intelligence quotient

**Figure 2:**
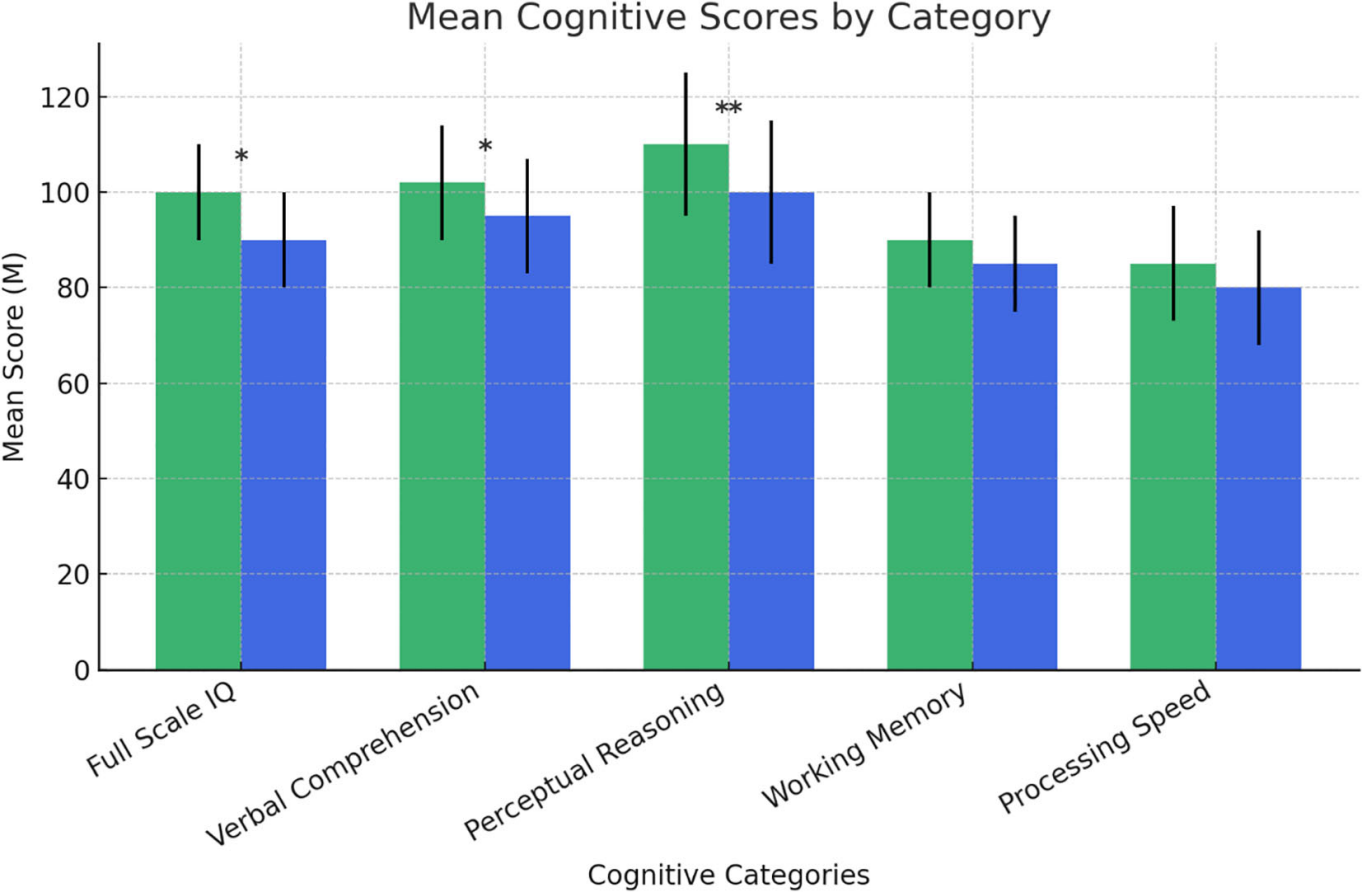
Intelligence Quotient tested by WISC-IV (NDDs at-risk *vs* no-rRisk). NDDs at-risk; no-risk. Statistically significant effects: (*): p < 0.05; (**): p < 0.01. Error bars for data variability. WISC-IV, Wechsler Intelligence Scale for Children – Fourth Edition (36).

**Figure 2** presents the comparison of intellectual functioning between the NDDs at-risk and no-risk groups, as measured by the Wechsler Intelligence Scale for Children – Fourth Edition (39). A significant difference was found in Full Scale IQ (F (1,84) = 4.802, p = .031), with the NDDs “at-risk group” scoring higher than the “no-risk group”.

Significant differences also emerged in specific cognitive domains. The NDDs “at-risk group” outperformed the “no-risk group” in Verbal Comprehension (F (1,84) = 4.048, p = .048) and Perceptual Reasoning (F (1,84) = 8.264, p = .005). No significant differences were observed in Working Memory (F (1,84) = 3.370, p = .070) or Processing Speed (F (1,84) = 0.828, p = .366), suggesting similar performance between the two groups in these cognitive domains.

These results are also shown in **Supplementary Table S2**.

### 3.4 Psychopathological profile

**Figure 3:**
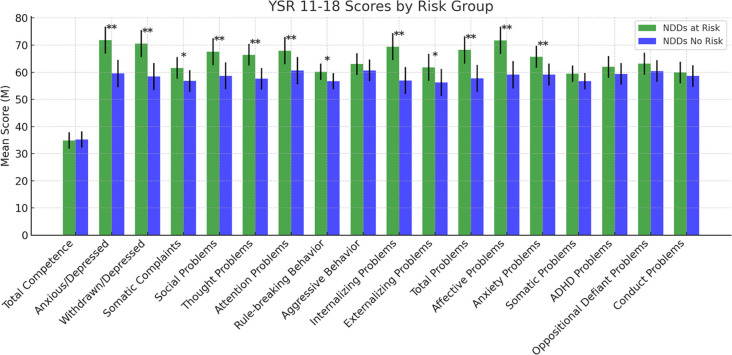
Scores at the Youth Self Report. Statistically significant effects: (*): p < 0.05; (**): p < 0.01. Error bars for data variability. YSR 11-18= Youth Self Report 11–18 (38).

**Figure 4:**
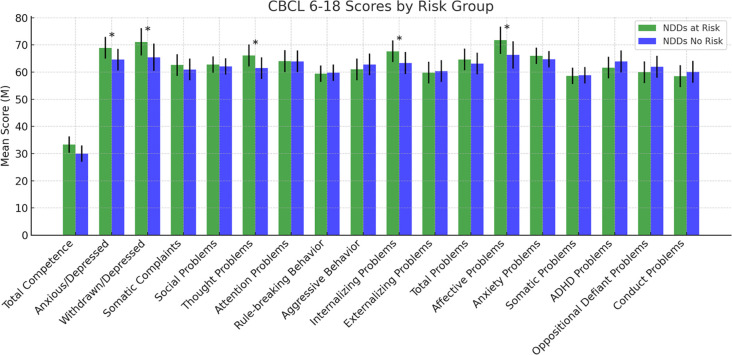
Scores at the Child Behavior Checklist. Statistically significant effects (*): p < 0.05. Error bars indicate data variability. CBCL 6–18 = Child Behavior Checklist 6–18 (38).

**Figure 5:**
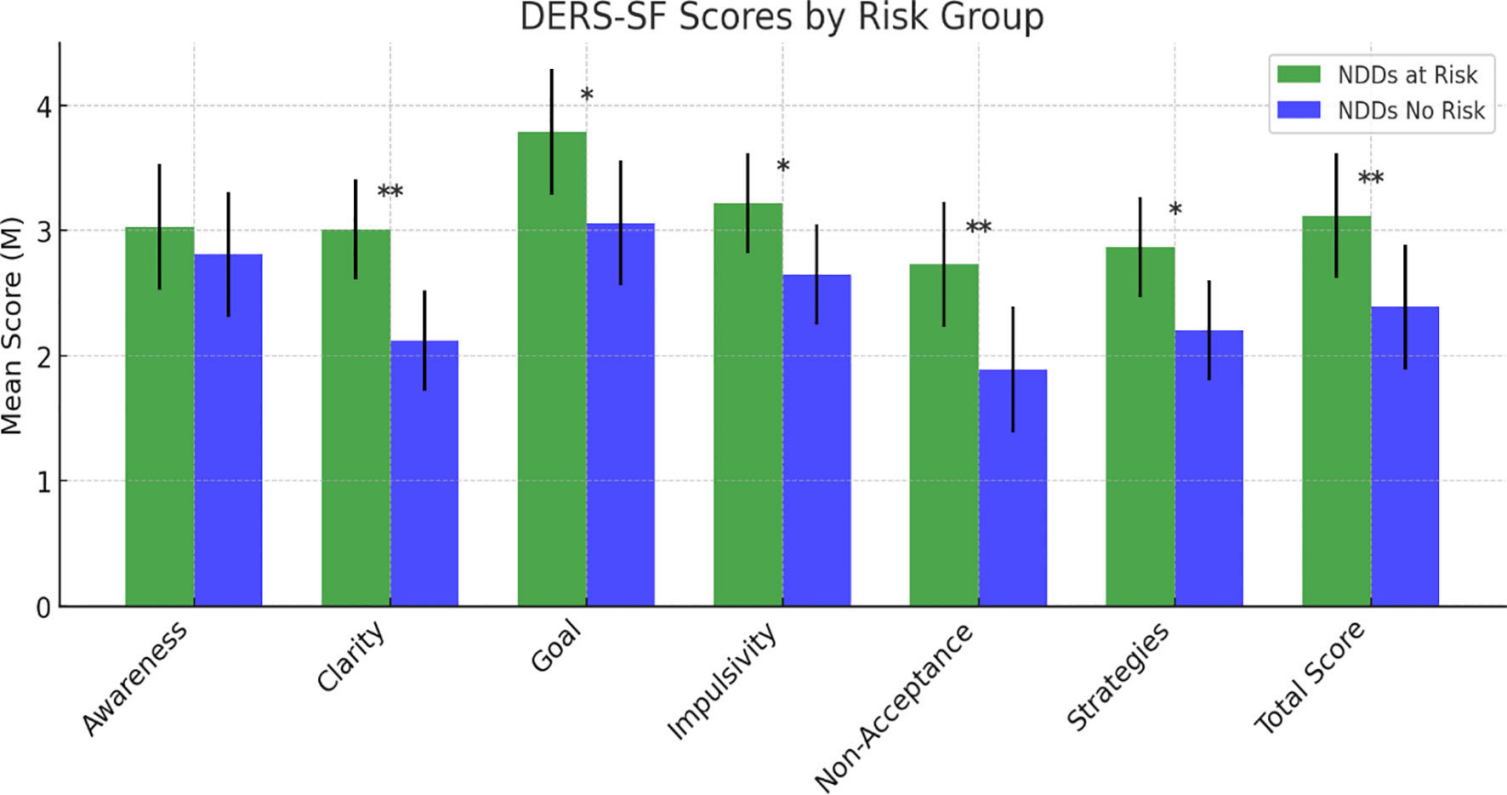
Scores at the Difficulties in Emotion Regulation Scale – Short Form. Statistically significant effects: (*): p < 0.05; (**): p < 0.01. Error bars for data variability. DERS-SF = Difficulties in Emotion Regulation Scale – Short Form (40).

**Figure 6:**
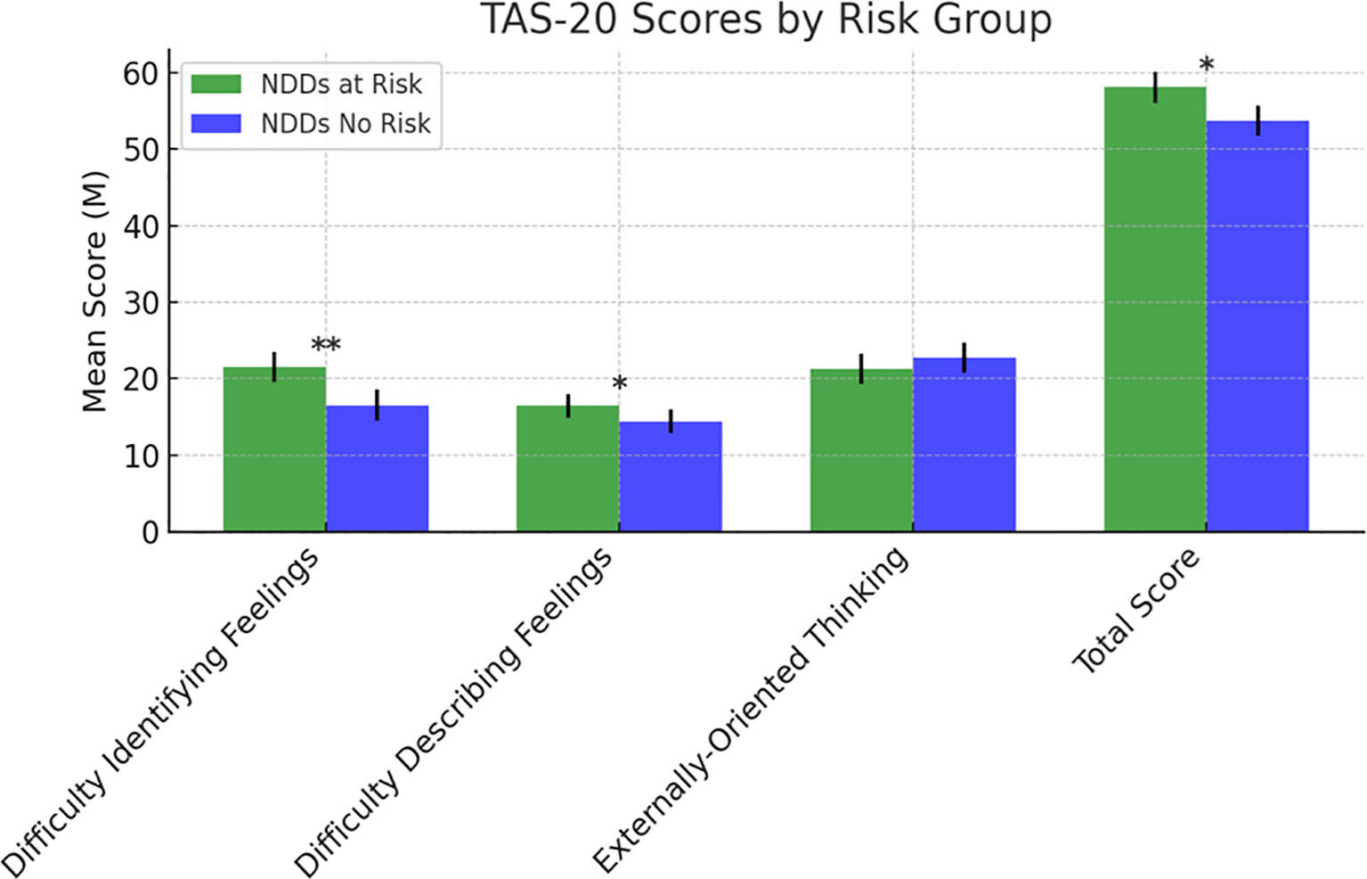
Scores at the Toronto Alexithymia Scale-20. Statistically significant effects: (*): p < 0.05; (**): p < 0.01. TAS-20, Toronto Alexithymia Scale-20 (41, 42).

**Figure 7:**
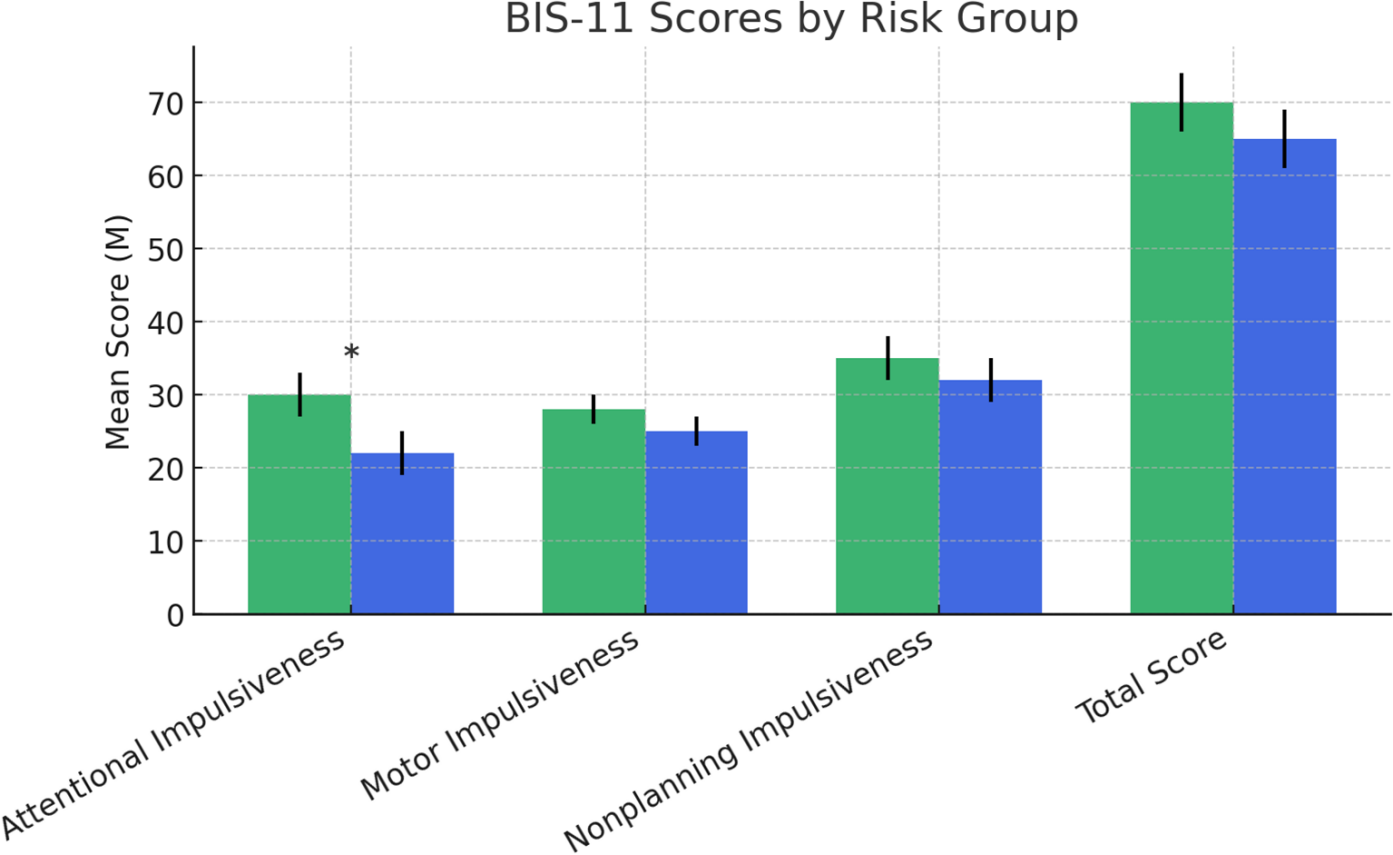
Scores at the Barratt Impulsiveness Scale-11. Statistically significant effects: (*): p < 0.05: p < 0.01. Error bars for data variability. BIS-11, Barratt Impulsiveness Scale-11 (43–45).

**Figure 8:**
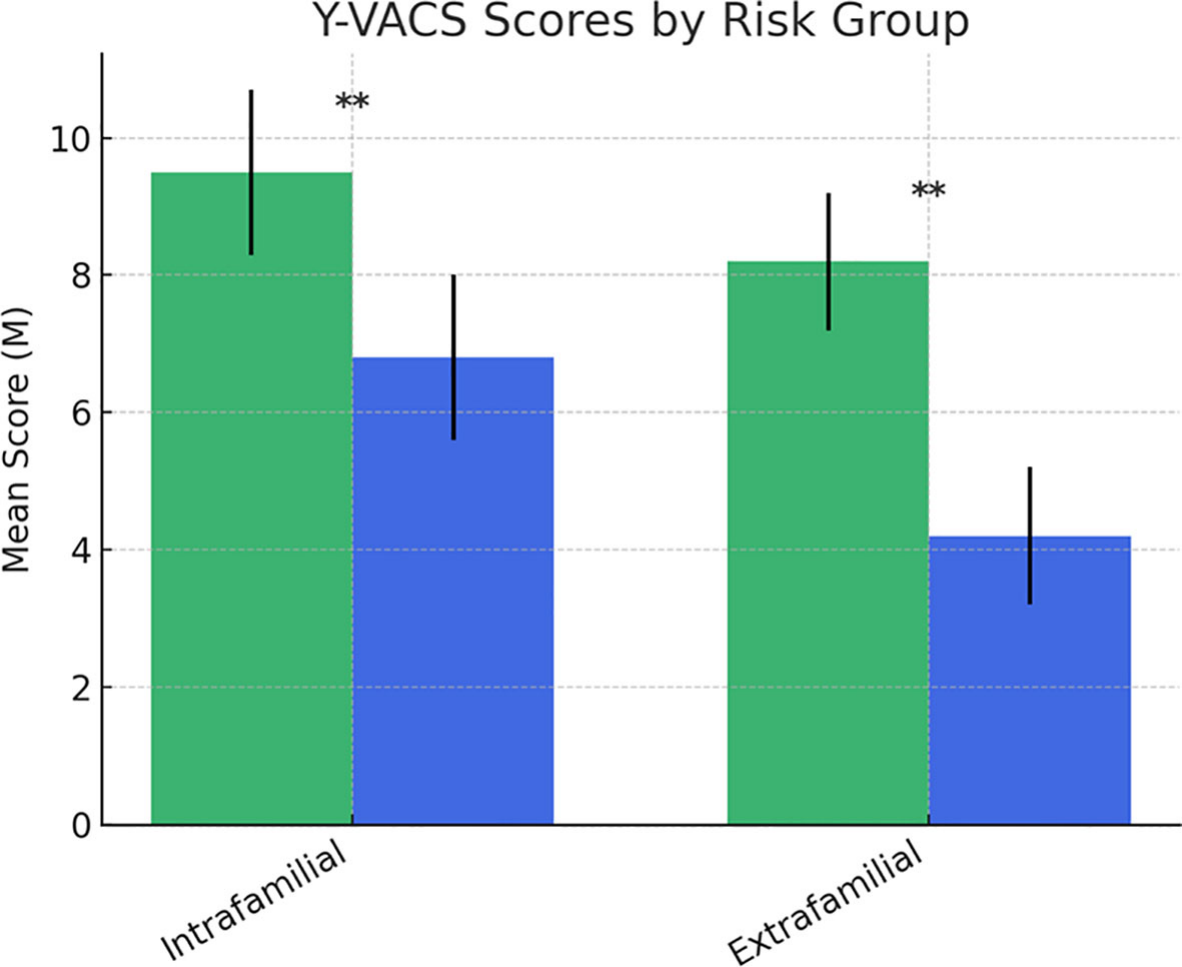
Scores at the Yale-Vermont Adversity in Childhood Scale. Statistically significant effects (**): p < 0.01. Error bars indicate data variability. YVACS, Yale-Vermont Adversity in Childhood Scale.

The differences in psychological variables between the “at-risk group” and “no-risk group” are shown in **Figures 3**–**8** and **Supplementary Tables S1–S3**.

Regarding the YSR 11-18, significant group differences were found for several subscales. The NDDs “at-risk group” showed higher scores compared to the NDDs “no-risk group” on Anxious/Depressed (F (1,126) = 33.273, p <.001), Withdrawn/Depressed (F(1,126) = 35.595, p <.001), Somatic Complaints (F(1,126) = 9.308, p = .003), Social Problems (F(1,126) = 25.948, p <.001), Thought Problems (F(1,126) = 28.972, p <.001), Attention Problems (F(1,126) = 12.447, p <.001), and Rule-breaking Behavior (F(1,126) = 4.744, p = .031). Furthermore, significant differences were observed for Internalizing Problems (F (1,126) = 37.976, p <.001), Externalizing Problems (F(1,126) = 6.257, p = .014), and Total Problems (F(1,126) = 27.281, p <.001). Affective Problems (F(1,126) = 34.871, p <.001) and Anxiety Problems (F(1,126) = 17.837, p <.001) also demonstrated significant differences, highlighting higher scores for the NDDs “at-risk group” compared to the “no-risk group”. However, no significant group differences emerged for the other subscales (**Figure 3** and **Supplementary Table S3**).

For the CBCL 6-18, significant group differences were found for Anxious/Depressed (F(1,126) = 5.176, p = .025), Withdrawn/Depressed (F(1,126) = 5.320, p = .023), Thought Problems (F(1,126) = 7.355, p = .008), Internalizing Problems (F(1,126) = 4.156, p = .044), and Affective Problems (F(1,126) = 8.529, p = .004), highlighting higher scores for the NDDs “at-risk group” compared to the “no-risk group”. No other significant differences emerged for the remaining subscales (**Figure 4**).

Regarding emotional dysregulation, as measured by the Difficulties in Emotion Regulation Scale – Short Form (DERS-SF) (43), significant group differences emerged in several subscales. The NDDs “at-risk group” reported higher scores on Clarity (F (1,126) = 19.832, p <.001), Goal (F (1,126) = 11.271, p = .001), Impulsivity (F (1,126) = 5.541, p = .020), Non-Acceptance (F (1,126) = 19.489, p <.001), and Strategies (F (1,126) = 11.560, p = .001). The total DERS-SF score was also significantly higher in the NDDs “at-risk group” compared to the “no-risk group” (F (1,126) = 22.076, p <.001. No significant differences were found in the Awareness subscale (F (1,126) = 1.336, p = .250) (**Figure 5**).

Regarding alexithymia, assessed using the Toronto Alexithymia Scale-20 (TAS-20) (43, 44), the NDDs “at-risk group” exhibited significantly greater difficulty in identifying feelings (F (1,126) = 16.660, p <.001) and difficulty describing feelings (F (1,126) = 5.210, p = .024). However, no significant differences emerged in externally oriented thinking (F (1,126) =2.052, p = .155). The total TAS-20 score was significantly higher in the NDDs “at-risk group” than in the “no-risk group” (F (1,126) = 4.080, p = .046) (**Figure 6**).

In terms of impulsivity, measured by the Barratt Impulsiveness Scale-11 (BIS-11) (46–48), a significant difference was found in attentional impulsiveness, with the NDDs at-risk group reporting higher scores compared to the no-risk group (F (1,126) = 3.815, p = .05). No significant differences were found for motor impulsiveness (F (1,126) = 0.510, p = .477), non-planning impulsiveness (F (1,126) = 1.791, p = .183), or the total BIS-11 score (F (1,126) = 0.022, p = .883) (**Figure 7**).

Finally, significant differences emerged in violence exposure, as measured by the Yale-Vermont Adversity in Childhood Scale (Y-VACS) (49). The NDDs “at-risk group” reported significantly higher exposure to intrafamilial adverse events compared to the “no-risk group” (F (1,126) = 9.648, p = .002). Similarly, exposure to extrafamilial adverse events was significantly higher in the NDDs “at-risk group” than in the “no-risk group” (F (1,126) = 9.819, p = .002) (**Figure 8**).

These results are also shown in **Supplementary Table S2**.

## 4 Discussion

The present study strongly supports the hypothesis that adolescents and pre-adolescents with NDDs at-risk for suicidal spectrum behaviors have a specific and predictive psychopathological profile compared to individuals with NDDs with no-risk for SSB. This comparative analysis of selected variables across NDDs groups covers the existent gap in literature on potential factors related to SSB that have not yet been extensively analyzed in this population.

In a previous narrative review by our group (9), we showed that emotion dysregulation (ED) may be considered an individual strong factor related to suicidality in subjects with NDDs. Nevertheless, we also found that adverse childhood experiences (ACE), as environmental factors, may promote suicidality in all individuals with NDD. Importantly, existing evidence suggests that individuals with NDDs are not only more frequently exposed to ACEs, but also more vulnerable to their psychological consequences compared to the general population. This increased susceptibility may contribute to the higher rates of suicidal ideation and behavior observed in these individuals (19, 51, 52). The present research strongly supports the theoretical model that we had proposed, in which both ED and ACE can lead to self-harm or suicide directly or indirectly by interacting with depressive symptoms. However, the broad array of information we collected on our sample allows us to add additional information. Indeed, the comparison between subjects with suicide-risk behaviors (“at-risk group”) and subjects without suicide-risk behaviors (“no-risk group”), segregated based on the Columbia-Suicide Severity Rating Scale (C-SSRS) scores, revealed a series of differences between the two groups. Screening for suicide risk in children and adolescents with NDDs represents significant challenges, mostly because there are no validated and customized tools and strategies. Over the past two decades, substantial efforts have focused on developing screening tools and strategies tailored specifically to youth, acknowledging the limitations of adult-focused models (53). However, concerns remain about the reliability of self-report questionnaires and interviews, which may be affected by cognitive biases or the transient nature of emotional states.

There is a lack of specific research on the efficacy of both psychotherapeutic interventions and psychopharmacological treatments in cohorts of individuals with NDDs and suicidality. The therapeutic approach is currently based on the general principles for intervention in pediatric population. Some psychotherapeutic approaches have garnered attention, including Integrated Cognitive-Behavioral Therapy (I-CBT), Multisystemic Therapy (MST), Mentalization-Based Treatment for Adolescents (MBT-A), Developmental Group Psychotherapy (DGP), the Resourceful Adolescent Parent Program (RAP-P), and Dialectical Behavior Therapy for Adolescents (DBT-A) (54). Pharmacological treatment has not proven to be more effective than psychotherapy or combined approaches in reducing suicidal outcomes (55). Nonetheless, current literature supports the use of medication. Since untreated depression is a major risk factor for suicide, the efficacy of antidepressants is well established, even though cautious during the use of SSRIs is recommended for the potential increasing effect on suicidality of these class of drugs in young individuals (56). Strong evidence in favor of anti-suicidal properties of Lithium treatment has been collected in the last decades (57) even though there is a lack of controlled studies in children and adolescents. Recent studies have investigated neurofeedback as a potential protective intervention against chronic depression, particularly in adolescents with neurodevelopmental disorders (NDDs) such as ADHD (58). Its therapeutic potential lies in its ability to modulate limbic circuitry (specifically the right amygdala, hippocampus, and anterior cingulate cortex) which is involved in depressive rumination, autobiographical memory, and implicit emotion regulation (59, 60).

### 4.1 Sociodemographic characteristics

Some of the differences observed between the “at-risk” and “no-risk” groups reflect broader trends already reported in the general adolescent population, particularly regarding age, gender, and the role of cognitive, social and environmental factors (61). Individuals at-risk are older than individuals belonging to the “no-risk group”. This data coincides with what has already been described for the general population of teenagers in which suicide is rare in childhood and early adolescence while it becomes more frequent with increasing age (62). Furthermore, this data should be referred to the concept of longitudinal trajectories of mental health problems in children with NDDs (63), prompting to pay attention since childhood to the factors that can promote a drift toward the adoption of suicidal behaviors later in life.

Gender distribution also varied significantly with a higher proportion of females in the NDDs “at-risk group” (63.3%) and of males in the “no-risk group” (63.5%). This finding is consistent with the data regarding the non-clinical population. A systematic review of population-based longitudinal studies (64), assessing associations between gender and suicide attempts/death in a non-clinical population (aged 12–26 years), showed a higher risk of suicide attempt in females (OR 1.96, 95% CI 1.54-2.50), but a higher risk of suicide death in males (HR 2.50, 95% CI 1.8-3.6). This may suggest paying close attention to at-risk males with NDDs who, although fewer in number, could be more likely to commit suicide.

Neither the socioeconomic status nor the immigration status seems to significantly impact suicide risk in our sample. This is somewhat consistent with the results of a recent systematic review (65) showing no major differences in suicidal ideation and suicide death in young migrants, even though they experience higher rates of self-harm and suicide attempts. However, these variables may be strongly related to the social context. To confirm this idea, in our sample, we found differences between different areas of our country. Participants from Emilia-Romagna (a region of northern Italy) were more frequently in the “at-risk group” (18.9%) rather than in the “no-risk group” (6.8%). We found the opposite in Sicily (a region of southern Italy), where the NDDs subjects predominantly belonged to the “no-risk group” (45.9%). These two areas of Italy differ in socio-economic status (higher in the north) (66) and predominant cultural models (larger and more supportive families and social groups in the south (67) Overall, a higher standard of living and a lower social cohesion seem to favor suicidality in adolescents with NDDs.

### 4.2 Family and individual medical history

Significant differences emerged between the “at-risk group” and the “no-risk group” in individual variables, while no differences emerged in psychiatric and medical family history. Therefore, individual variables seem to have a more significant impact on suicidal risk rather than family variables.

First of all, individuals belonging to the “at-risk group” reported a higher prevalence of substance abuse (15.7%) compared to the “no-risk group” (4.1%). This data was somewhat expected given the large amount of literature data on the general population that correlates the use of psychoactive substances in adolescents with an increased risk of suicide (68). Suicidality and problematic substance use are, therefore, strictly linked. Also, neurodevelopmental disorders and addiction are strongly correlated to the point that some authors [e.g. (69)] have proposed to view addiction as a neurodevelopmental disorder. They argue that the underpinned structural and/or functional alterations of the brain regions controlling emotion, reasoning, language, and memory make individuals with NDDS more prone to develop a problematic substance use.

Secondly, the risk of suicide appears to increase with increasing complexity and severity of the clinical phenotype. Both hospitalizations and treatments (psychopharmacological and psychological) were significantly more frequent among the NDDs “at-risk group” (43.1%) compared to the “no-risk group” (21.7%). Additionally, ongoing treatment was significantly more common in the NDDs “at-risk group” accounting for the persistence of symptoms and the higher resistance to treatment.

Among the psychiatric diagnoses associated with NDDs, major depression was significantly more represented in the NDDs “at-risk group” (30.8%) than in the “no-risk group” (6.8%). This is fully coherent with the existing data describing depressive symptoms as the main psychological issue associated with suicide, both in individuals with neurodevelopmental disorders (9) and in the general population (70). It is widely demonstrated that children with NDDs are at increased risk of developing depression. Irritability seems to be the symptom more strongly connected with later depression (71). After all, the link between NDDs and depression has long been described as probably causal. In particular, it has been argued that certain vulnerability factors affecting the maturation of brain circuits result in emotional dysfunction and lead to an increased risk for depressive disorders later in life (72).

Significantly, in our sample, the rate of bipolar disorder (BD) diagnosis does not reach the statistical significance between at-risk and no-risk groups, even though BD is more frequent in the NDDs “at-risk group” (13.5%) than in the “no-risk group” (5.5%). This finding does not appear consistent with literature data on general population showing that the 25% of pediatric population with BD is at high risk of attempting suicide, 50% has suicidal ideation (73) and 18% reports attempting suicide in a five-year longitudinal follow up (74). Furthermore, the effect of bipolar experiences on suicide risk seems to be mediated by behavioral and emotional difficulties closely associated with NDDs (75). We postulate that the lack of statistical significance between at-risk and no-risk groups, in our sample, is due to the underdiagnosis of BD, even more frequent when BD is associated to ADHD or other MDDs (76).

### 4.3 Intelligence quotient

A significant difference was found in Full Scale IQ at WISC-IV, with the individuals at-risk scoring higher than those of the “no-risk group”. An opposite relationship is described in the general population where a graded association between lower childhood IQ and suicide attempt has been reported in large cohort studies (77). In our sample, significant differences also emerged in two of four derived indices from the Wechsler Intelligence Scale for Children, “Verbal Comprehension” (VCI) and “Perceptual Reasoning” (PRI), with the NDDs “at-risk group” outperformed the “no-risk group”. Both VCI and PRI measure the reasoning skills applied to word knowledge and to nonverbal information, accounting for the child’s ability to understand his environment, express himself in a meaningful manner, examine novel problems, organize thoughts, examine rules and logical relationships, and create adaptive solutions. Surprisingly, these abilities appear to constitute a disadvantage in terms of suicidal risk in the group of subjects with NDDs that we have examined.

To our knowledge, there are no previous data in the literature describing the association between IQ and suicide risk in a heterogeneous population of subjects with NDDs. The only available data concern the ASD population in which the “vulnerability effect” of intelligence is already described. In fact, an increased risk of suicide has been reported in ASD individuals with a higher intelligence quotient compared to those with a lower IQ (78). It has been argued that adolescents with ASD without intellectual disabilities are more at-risk for depressive symptoms and SSB due to a clearer awareness of their interpersonal difficulties associated with social isolation and exclusion (78, 79).

It should be emphasized that there may be numerous confounding factors when examining the relationship between IQ and suicide risk. For example, in a Swedish large population-representative cohort, in which lower IQ predicted subsequent suicide attempts, potential confounding factors have been analyzed, revealing that poor academic performances at age 16 were a robust predictor of suicide attempts in young adulthood (77). On the contrary, even in high-functioning ASD individuals at high risk for suicide, solid social relationships count as a protective factor, even in the presence of depressive symptoms (78).

### 4.4 Psychopathological profile

Large statistical differences emerged in psychopathological variables between the NDDs at-risk and those with no-risk for SSB, accounting for the presence of specific warning signs for suicide risk in the NDDs population.

Starting from the self-report scale YSR 11-18, we can draw a psychopathological profile of subjects at-risk, mainly characterized by internalized symptoms (affective problems, anxious and depressive symptoms, frequent somatic complaints) and social and cognitive difficulties (thought and attention problems). Externalizing symptoms are significantly less represented since no differences emerged in aggressive behaviors and oppositional/defiant and conduct symptoms. Interestingly, the at-risk subjects show more frequent rule-breaking behaviors. This variable, often perceived as defiance or rebellion against norms, is actually influenced by several factors, ranging from cultural and social instances to psychological dimensions and including coping strategies against stress, anxiety and emotional concerns (e.g. refuse or avoiding to do something), frequently associated with NDDs (80). The findings from the CBCL 6-18, administered to parents, largely confirm the evidence from YSR 11–18 showing significant group differences for internalizing problems, such as anxious/depressive symptoms, withdrawal behaviors, and affective problems, as well as for thought problems. On the contrary, no significant differences in the subscales measuring the externalizing symptoms emerged from the comparison between at-risk and no-risk participants. Overall, the results arising from the YSR 11–18 and CBCL 6–18 lead to the conclusion of the central role of internalizing symptoms in supporting suicidal spectrum behaviors in NDDs individuals. These findings are in contrast with the studies that link the adolescent self-harm ideation and behaviors to the so called “CBCL–Dysregulation Profile (DP), a mixed phenotype characterized by both internalizing (anxiety/depression) and externalizing (attention deficit/hyperactivity and aggression) symptoms (81). This difference suggests that, among youth with NDDs, the affective component rather than the behavioral one may be prominent in increasing the risk of suicide.

Regarding emotional dysregulation, as measured by DERS-SF, the differences between the two groups are highly significant, suggesting poorer ability to regulate the intensity and quality of emotions in at-risk NDDs subjects. The relationship between ED and suicidality has been widely described in the literature with the idea that the poor ability to regulate emotions can lead to consider SSB as a possible strategy to regulate emotional levels and to escape emotional suffering (82). Especially for this reason, ED has been described as an independent risk-increasing factor for suicidal ideation, even among adolescents [e.g. (83)]. Moreover, ED has been conceptualized as a transdiagnostic feature in young individual NNDs (84). We studied our sample using DERS, a rating scale based on a multidimensional model of emotion regulation and describing emotion dysregulation as a maladaptive response to feelings of distress. In our sample, almost all six DERS dimensions were more impaired in at-risk participants compared to no-risk individuals. Both groups showed a lack of emotional awareness and an unwillingness to acknowledge emotions, as expected in young people with NDDs. Nevertheless, the at-risk group was particularly impaired in the dimension “Clarity” (difficulty in clearly recognizing emotional experiences and distinguishing between different emotions felt) and “Non Acceptance” (difficulty in accepting ones’ emotions with the tendency to experience secondary emotions in response to those not accepted). The difficulty in recognizing and accepting emotions is a widespread condition among young people with NDDs, and it is not at all surprising to find that as this condition worsens, the risk of suicide increases. However, based on the differences that emerged between the two groups, these two dimensions could be considered the main emotional risk indicators among young people with NDDs. A clear recognition of emotional experiences, together with the ability to accept even the negative ones, seem to be crucial for regulating the emotional response. This is consistent with previous literature data (85) showing that in adolescent inpatients, the limited ability to access emotion regulation strategies, assessed by DERS, is significantly associated with suicidal ideation and attempts.

By the TAS-20, we assessed alexithymia, finding that the NDDs at-risk group had greater difficulty in identifying and describing feelings than the no-risk group. Frequently described in ASD, but also very common among children and adolescents with other NDDs, Alexithymia can be considered a transdiagnostic construct (86, 87) consisting of the difficulty in identifying and describing feelings. Consistent evidence demonstrates an association between alexithymia and SSB both in adults and in young people, even though depression probably accounts for part of the relationship between alexithymia and suicidality (88). Alexithymia predicts the risks for psychopathology and SSB, especially during the transition from childhood to adolescence (89). In depressed adolescents, Alexithymia may have an impact on self-harming behavior associated with somatization symptoms, the body being used to express emotional issues (90). In light of these evidence, the difficulties in expressing feelings in a verbal/semantic form should be carefully evaluated, especially in NDDs population, as a strong risk factor for SSB since it reduces the capacity to understand and regulate emotions.

Impulsivity is a long-known risk factor for suicidal behaviors (91) and, at the same time, it is associated with several psychiatric disorders, including ADHD, ASD, and other NDDS (9). We studied impulsiveness in our sample using the BIS-11, a “multifaceted” measure of three main impulsiveness domains: attention impulsivity, motor impulsivity, and nonplanning impulsivity. The NDDs at-risk group was significantly different from the no-risk group only in the first domain. “Attentional Impulsiveness” sub-scale assesses task-focus, and intrusive/racing thoughts, a sort of mental restlessness that can interfere with daily activities. Interestingly, some research on SSB shows a failure of higher-order control and the “decision-making” process in the context of impulsivity in the suicidal mind (92). Also, in young people, the deficit in impulsive decision-making has been associated with self-harm or suicidal behavior (93). In our sample, just this domain appears a discriminant element between at-risk and no-risk NDDs subjects. It could be related to the presence of ruminative thoughts about death and suicide associated with difficulties in maintaining focus or acting without forethought that may interfere with the appropriateness of decision making. This is consistent with previous data on community-based adolescents (94) showing that the cognitive facets of impulsivity are associated with the maintenance of self-harm, differently from the mood-based impulsivity related to the initiation of self-harm. For example, in our sample, neither Motor Impulsiveness (tendency to act on the spur of the moment) nor Non-planning Impulsiveness (focusing on the present moment without regard for future consequences) is related to the SSB risk. Overall, these elements support the central role of the cognitive facets of impulsivity in improving the suicide risk in individuals with NDDs.

**Figure 9:**
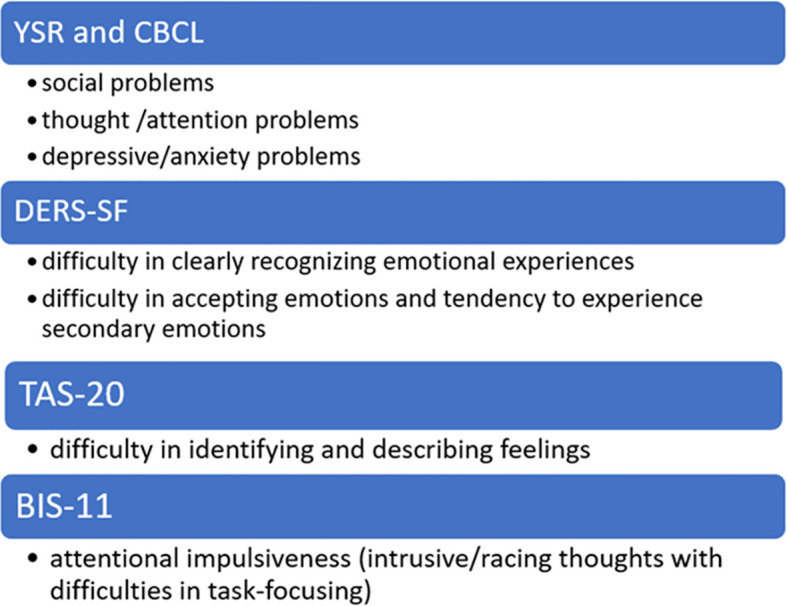
Psychological Red Flags for suicide risk in NDDs sample.

**Figure 9** shows a synthesis of the main psychological domains related to SSB risk and assessed by the mentioned scales in the enrolled sample of youth with NDDs. We focused our attention on some variables that can be considered as red flags for the suicide risk in this population. The listed variables have been inferred by the present research and cannot be considered universally valid for the whole NDDs population. They may represent a suggestion to pay attention to some elements that strongly increase the suicide risk in adolescents and pre-adolescents with NDDs.

### 4.5 Adverse childhood experiences

In a previous narrative review by our group (9), ACEs (Adverse Childhood Experiences) have been identified as a strong factor in promoting suicide in population with NDDs. We proposed a theoretical model in which ACEs, together with emotion dysregulation, directly or indirectly by interacting with depressive spectrum disorders, promoted SSB. Empirical data collected by the present research largely confirmed this assumption. In our sample, significant differences emerged in violence exposure, as measured by the Y-VACS, between at-risk and no-risk adolescents and pre-adolescents with NDDs. Significantly, higher exposure to both intrafamilial adverse events and extrafamilial adverse events was found in the at-risk group compared to the no-risk group. This is widely expected since strong evidence links ACEs to an increased risk of suicidal behaviors also in the general pediatric population (95). Few studies have studied the link between NDDs and suicidal risk. A recent, observational, prospective, single-center study (96) found higher traits indicative of NDDs in a sample of children and adolescents hospitalized for a serious suicide attempt compared to the general pediatric population. More in detail, 70% of the participants had at least one neurodevelopmental disorder (autistic traits, ADHD, learning disorder, or motor disorder), and 65% of them had at least one behavioral disorder (oppositional defiant disorder, conduct disorder). In parallel, an increased risk of experiencing ACEs in the population of subjects with NDDs has been described (97). Since having NDDs increases per se the risk of suicide and ACEs do the same, one might hypothesize the presence of a cumulative risk for subjects with both NDDs and a higher burden of ACEs. In any case, the overall data suggests considering the presence of ACEs in subjects with NDDs as a further red flag for the risk of suicide.

## 5 Limitations

There were some limitations to this study. First, due to the single time point of data collection and the lack of longitudinal data, the applied model could appear too simple. Further studies with more time points could bring to light more complex trajectories. Due to the relatively small sample size, it was not possible to quantify the odds ratio for each risk factor and neither to describe the specific risk factors for each NDDs. Moreover, the population of subjects with NNDDs has not been compared with a non-clinical population sample. Additionally, the psychopathological and behavioral symptoms have been mainly explored by caregivers and self-report scales. This approach could have caused some false negatives because adolescents are sometimes reluctant to reveal their suicidal thoughts and behaviors and even parents are unaware of their children’s thoughts. Nevertheless, all subjects in the sample are regularly followed up at the centers where they were recruited. This mitigates biases related to both the lack of multiple time points and the limitation of scales as diagnostic tools.

## 6 Future perspectives

The present research suggests future projects to be further explored. First, a comparison between the general pediatric population and a sample of individuals with NDDs would be appropriate in order to identify similarities and differences in suicide risk factors. A peculiar route to suicidality has been postulated for all NDDs, although very few studies systematically investigated this issue so far. It would be interesting to identify risk factors for suicidality specifically for each NDD. For instance, it is postulated that “high intellectual functioning” and “camouflaging” may raise the suicide risk in ASD population (9). But very few clinical studies have proved this postulate. Another interesting topic related to suicidality in NDD would be the gender differences, since specific risk/protective factors of suicide death in adolescent and young adult females, compared to males, have been described (64). Finally, it would be of great help in clinical practice to have standardized tools for the screening and the diagnosis of suicidality in this specific population.

## 7 Conclusions

A specific psychological and socio-demographic profile as a route to suicidality has been broadly postulated for individuals with NDDs, although this issue has not been broadly investigated so far. Even if preliminary, this research provides useful data to identify the at-risk individuals. Clinicians and caregivers should be fully aware that having an NDDs increases the risk of dying by suicide.

The possibility of scotomizing risk signals is higher in the population of subjects with NDDs and due to many reasons. First of all, the limited ability of young people with NDDs to conceptualize and translate pain into words. Secondly, the “halo effect” on clinicians and caregivers who are focused on the symptoms of the neurodevelopmental disorder and may neglect any other psychiatric symptoms. Finally, the poor knowledge of the red flags in this specific population of individuals. The main purpose of this study is to stimulate the expansion of this research area. Understanding which risk factors negatively impact mental health in individuals with NDDs is crucial for improving the primary prevention of suicide and promoting the protective factors associated with ending suicide.

## Data Availability

The raw data supporting the conclusions of this article will be made available by the authors, without undue reservation.
