# Supplementary material for: Suicidal risk and psychopathological profiles in adolescents with neurodevelopmental disorders: an Italian multicentric study
Source: Front Psychiatry. 2025 Aug 15;16:1614270. doi: 10.3389/fpsyt.2025.1614270 (PMC12394981; doi:10.3389/fpsyt.2025.1614270)
Supplement: Supplementary file 1 [file Table1.docx]

Supplementary Material

# Supplementary Tables

**Table S1** **Family and Individual Variables: NDDs group at-risk vs no-risk**

| **Variable** | **N** | **NDDs at-risk**  **% (N)** | **NDDs no-risk**  **% (N)** | ***χ²*** | ***p*** |
| --- | --- | --- | --- | --- | --- |
| **Psychiatric Family History** |  |  |  | 0.107 | .744 |
| Yes | 61 | 62.0 (31) | 58.8 (30) |  |  |
| No | 40 | 38.0 (19) | 41.2 (21) |  |  |
| **Family History of Health Problems** |  |  |  | 0.958 | .328 |
| Yes | 47 | 52.1 (25) | 42.3 (22) |  |  |
| No | 53 | 47.9 (23) | 57.7 (30) |  |  |
| **Chronic Medical Conditions** |  |  |  | 0.316 | .574 |
| Yes | 10 | 9.6 (5) | 6.8 (5) |  |  |
| No | 115 | 90.4 (47) | 93.2 (68) |  |  |
| **Neurological Conditions** |  |  |  | 0.136 | .712 |
| Yes | 11 | 7.7 (4) | 9.6 (7) |  |  |
| No | 114 | 92.3 (48) | 90.4 (66) |  |  |
| **Substance Abuse** |  |  |  | 5.090 | **.024** |
| Yes | 11 | 15.7 (8) | 4.1 (3) |  |  |
| No | 114 | 84.3 (43) | 95.9 (71) |  |  |
| **Previous Hospitalizations** |  |  |  | 6.296 | **.012** |
| Yes | 37 | 43.1 (22) | 21.7 (15) |  |  |
| No | 83 | 56.9 (29) | 78.3 (54) |  |  |
| **Neuropsychiatric and Psychological Interventions** | | | | 7.812 | **.005** |
| Yes | 79 | 82.0 (41) | 57.6 (38) |  |  |
| No | 37 | 18.0 (9) | 42.4 (28) |  |  |
| **Ongoing Psychopharmacological Treatment** |  |  |  | 13.095 | **<.001** |
| Yes | 65 | 76.0 (38) | 42.2 (27) |  |  |
| No | 49 | 24.0 (12) | 57.8 (37) |  |  |
| **Major Depression** |  |  |  | 12.431 | **<.001** |
| Yes | 21 | 30.8 (16) | 6.8 (5) |  |  |
| No | 104 | 69.2 (36) | 93.2 (68) |  |  |
| **Bipolar Disorder** |  |  |  | 2.411 | .12 |
| Yes | 11 | 13.5 (7) | 5.5 (4) |  |  |
| No | 114 | 86.5 (45) | 94.5 (69) |  |  |

*Note.* The values presented in bold indicate statistically significant effects

**Table S2 Intelligence Quotient: NDDs risk group vs no-risk**

| **Variable** | **NDDs at-risk**  **M (SD)** | **NDDs no-risk**  **M (SD)** | ***F* (1, 84)** | ***p*** |
| --- | --- | --- | --- | --- |
| **WISC-IV** | | | | |
| Full Scale IQ | 102.60 (17.97) | 93.55 (20.10) | 4.802 | **.031** |
| Verbal Comprehension | 102.60 (17.97) | 99.12 (19.59) | 4.048 | **.048** |
| Perceptual Reasoning | 111.10 (16.55) | 99.59 (19.58) | 8.264 | **.005** |
| Working Memory | 92.76 (19.20) | 85.83 (14.66) | 3.370 | .070 |
| Processing Speed | 93.51 (24.52) | 89.24 (17.34) | 0.828 | .366 |

*Note.* The values presented in bold indicate statistically significant effects.

WISC-IV = Wechsler Intelligence Scale for Children – Fourth Edition (Wechsler, 2012)

**Table S3 Scores on Psychopathological Variables: NDDs group at-risk vs no-risk**

| **Variable** | **NDDs at-risk**  **M (SD)** | **NDDs no-risk**  **M (SD)** | ***F*(1, 126)** | ***p*** |
| --- | --- | --- | --- | --- |
| **YSR 11-18** | | | | |
| Total Competence | 34.86 (13.27) | 35.20 (13.01) | 0.018 | .894 |
| Anxious/Depressed | 71.82 (13.82) | 59.54 (9.51) | 33.273 | **<.001** |
| Withdrawn/Depressed | 70.58 (12.68) | 58.44 (9.60) | 35.595 | **<.001** |
| Somatic Complaints | 61.60 (9.20) | 56.81 (7.91) | 9.308 | **.003** |
| Social Problems | 67.56 (10.36) | 58.69 (8.67) | 25.948 | **<.001** |
| Thought Problems | 66.46 (9.74) | 57.61 (8.21) | 28.972 | **<.001** |
| Attention Problems | 67.96 (13.66) | 60.59 (9.24) | 12.447 | **<.001** |
| Rule-breaking Behavior | 60.16 (8.88) | 56.71 (8.30) | 4.744 | **.031** |
| Aggressive Behavior | 63.04 (10.53) | 60.69 (11.31) | 1.338 | .250 |
| Internalizing Problems | 69.44 (10.62) | 56.94 (11.18) | 37.976 | **<.001** |
| Externalizing Problems | 61.80 (10.67) | 56.27 (12.76) | 6.257 | **.014** |
| Total Problems | 68.22 (9.53) | 57.77 (11.63) | 27.281 | **<.001** |
| Affective Problems | 71.76 (11.73) | 59.09 (11.49) | 34.871 | **<.001** |
| Anxiety Problems | 65.72 (8.89) | 59.14 (8.06) | 17.837 | **<.001** |
| Somatic Problems | 59.50 (10.26) | 56.66 (8.32) | 2.802 | .097 |
| Attention Deficit Hyperactivity Problems | 61.98 (8.26) | 59.40 (7.88) | 3.005 | .086 |
| Oppositional Defiant Problems | 63.18 (9.57) | 60.46 (9.65) | 2.338 | .129 |
| Conduct Problems | 59.90 (9.61) | 58.64 (9.93) | 0.480 | .490 |
| **CBCL 6-18** | | | | |
| Total Competence | 33.39 (11.16) | 30.00 (8.27) | 3.416 | .067 |
| Anxious/Depressed | 68.92 (10.31) | 64.56 (10.17) | 5.176 | **.025** |
| Withdrawn/Depressed | 71.10 (14.27) | 65.46 (12.18) | 5.320 | **.023** |
| Somatic Complaints | 62.60 (9.09) | 60.96 (9.25) | 0.916 | .340 |
| Social Problems | 62.75 (8.02) | 62.09 (8.43) | 0.184 | .669 |
| Thought Problems | 66.10 (9.40) | 61.46 (8.97) | 7.355 | **.008** |
| Attention Problems | 64.02 (9.34) | 63.94 (9.53) | 0.002 | .965 |
| Rule-breaking Behavior | 59.40 (6.90) | 59.73 (8.34) | 0.052 | .820 |
| Aggressive Behavior | 61.00 (8.43) | 62.79 (9.76) | 1.062 | .305 |
| Internalizing Problems | 67.63 (9.42) | 63.34 (12.43) | 4.156 | **.044** |
| Externalizing Problems | 59.80 (8.43) | 60.39 (10.90) | 0.101 | .751 |
| Total Problems | 64.63 (9.14) | 63.13 (9.98) | 0.702 | .404 |
| Affective Problems | 71.73 (10.20) | 66.31 (9.68) | 8.529 | **.004** |
| Anxiety Problems | 66.02 (7.24) | 64.69 (8.36) | 0.809 | .370 |
| Somatic Problems | 58.65 (9.01) | 58.87 (9.61) | 0.017 | .898 |
| Attention Deficit Hyperactivity Problems | 61.63 (7.63) | 63.90 (9.05) | 2.037 | .156 |
| Oppositional Defiant Problems | 59.94 (6.42) | 61.94 (8.50) | 1.918 | .169 |
| Conduct Problems | 58.48 (7.51) | 60.10 (8.99) | 1.054 | .307 |
| **DERS-SF** | | | | |
| Awareness | 3.03 (1.08) | 2.81 (1.01) | 1.336 | .250 |
| Clarity | 3.01 (1.22) | 2.12 (1.00) | 19.832 | **<.001** |
| Goal | 3.79 (1.21) | 3.06 (1.19) | 11.271 | **.001** |
| Impulsivity | 3.22 (1.36) | 2.65 (1.29) | 5.541 | **.020** |
| Non-Acceptance | 2.73 (1.13) | 1.89 (0.99) | 19.489 | **<.001** |
| Strategies | 2.87 (1.25) | 2.20 (0.93) | 11.560 | **.001** |
| Total score | 3.12 (0.97) | 2.39 (0.78) | 22.076 | **<.001** |
| **TAS-20** | | | | |
| Difficulty Identifying Feelings | 21.50 (7.10) | 16.53 (6.47) | 16.660 | **<.001** |
| Difficulty Describing Feelings | 16.44 (4.95) | 14.45 (4.75) | 5.210 | **.024** |
| Externally-Oriented Thinking | 21.27 (7.01) | 22.74 (4.54) | 2.052 | .155 |
| Total score | 58.13 (14.25) | 53.72 (10.31) | 4.080 | **.046** |
| **BIS-11** | | | | |
| Attentional impulsiveness | 20.24 (4.32) | 18.64 (4.57) | 3.815 | **.05** |
| Motor impulsiveness | 22.51 (5.34) | 23.26 (6.04) | 0.510 | .477 |
| Nonplanning impulsiveness | 27.65 (5.18) | 28.81 (4.43) | 1.791 | .183 |
| Total score | 70.39 (11.35) | 70.70 (11.48) | 0.022 | .883 |
| **Y-VACS** | | | | |
| Intrafamilial | 9.44 (5.23) | 6.70 (4.32) | 9.648 | **.002** |
| Extrafamilial | 8.21 (8.12) | 4.13 (6.03) | 9.819 | **.002** |

*Note.* The values presented in bold indicate statistically significant effects.

YSR 11-18= Youth Self Report 11-18 (Achenbach, 2001); CBCL 6-18= Child Behavior Checklist 6-18 (Achenbach, 2001); DERS-SF = Difficulties in Emotion Regulation Scale – Short Form (Mancinelli et al., 2024); TAS-20 = Toronto Alexithymia Scale-20 (Bressi et al., 1996; Rieffe et al., 2006); BIS-11 = Barratt Impulsiveness Scale-11 (Fossati et al., 2001; Patton et al., 1995; Stanford et al., 2009); Y-VACS = Yale-Vermont Adversity in Childhood Scale (Hudziak & Kaufman, 2014).
